# Prognostic and predictive impact of stroma cells defined by PDGFRb expression in early breast cancer: results from the randomized SweBCG91RT trial

**DOI:** 10.1007/s10549-021-06136-4

**Published:** 2021-03-04

**Authors:** Carina Strell, Axel Stenmark Tullberg, Reidunn Jetne Edelmann, Lars Andreas Akslen, Per Malmström, Mårten Fernö, Erik Holmberg, Arne Östman, Per Karlsson

**Affiliations:** 1 Department of Oncology-Pathology, Karolinska Institutet, Karolinska University Hospital, Stockholm, Sweden; 2 Department of Oncology, Sahlgrenska University Hospital, Institute of Clinical Sciences, Sahlgrenska Academy, University of Gothenburg, Gothenburg, Sweden; 3 Department of Clinical Medicine, Centre for Cancer Biomarkers CCBIO, University of Bergen, Bergen, Norway; 4 Division of Oncology and Pathology, Department of Clinical Sciences, Lund University, Lund, Sweden; 5 Department of Haematology, Oncology and Radiation Physics, Skåne University Hospital, Lund, Sweden

**Keywords:** Early breast cancer, Radiotherapy, Tumor microenvironment, PDGFR, Fibroblasts

## Abstract

**Purpose:**

Predictive biomarkers are needed to aid the individualization of radiotherapy (RT) in breast cancer. Cancer-associated fibroblasts have been implicated in tumor radioresistance and can be identified by platelet-derived growth factor receptor-beta (PDGFRb). This study aims to analyze how PDGFRb expression affects RT benefit in a large randomized RT trial.

**Methods:**

PDGFRb was assessed by immunohistochemistry on tissue microarrays from 989 tumors of the SweBCG91RT trial, which enrolled lymph node-negative, stage I/IIA breast cancer patients randomized to RT after breast-conserving surgery. Outcomes were analyzed at 10 years for ipsilateral breast tumor recurrence (IBTR) and any recurrence and 15 years for breast cancer specific death (BCSD).

**Results:**

PDGFRb expression correlated with estrogen receptor negativity and younger age. An increased risk for any recurrence was noted in univariable analysis for the medium (HR 1.58, CI 95% 1.11–2.23, *p* = 0.011) or PDGFRb high group (1.49, 1.06–2.10, *p* = 0.021) compared to the low group. No differences in IBTR or BCSD risk were detected. RT benefit regarding IBTR risk was significant in the PDGFRb low (0.29, 0.12–0.67, *p* = 0.004) and medium (0.31, 0.16–0.59, *p* < 0.001) groups but not the PDGFRb high group (0.64, 0.36–1.11, *p* = 0.110) in multivariable analysis. Likewise, risk reduction for any recurrence was less pronounced in the PDGFRb high group. No significant interaction between RT and PDGFRb-score could be detected.

**Conclusion:**

A higher PDGFRb-score conferred an increased risk of any recurrence, which partly can be explained by its association with estrogen receptor negativity and young age. Reduced RT benefit was noted among patients with high PDGFRb, however without significant interaction.

## Introduction

Radiotherapy (RT) in combination with breast conserving surgery (BCS) is currently the preferred treatment over mastectomy for patients with early stage breast cancer. Nevertheless, a minority of these patients will suffer from local recurrences during the first decade after surgery [1, 2]. Classic histopathological variables are unable to identify patients with different proportional benefits from adjuvant RT [2]. An increasing focus is being put on the microenvironment as a modulator of the benefit from adjuvant RT. Recently, a high number of tumor-infiltrating lymphocytes was shown to independently reduce the recurrence risk of early stage breast cancer patients within a randomized RT trial. Consequently, patients with low levels of tumor-infiltrating lymphocytes may represent a subgroup with an increased RT benefit [3]. Preclinical studies have indicated that stroma cells can modulate radiosensitivity of tumor cells [4–7], but non-leukocytic stroma cells have not yet been explored as potential predictive markers for benefit of RT in invasive breast cancer.

Platelet-derived growth factor receptor beta (PDGFRb) is a key regulator of fibroblasts, pericytes and smooth muscle cells (reviewed in [8–10]). The role of stromal PDGFRb expression in progression and treatment response of invasive breast cancer is still not fully understood. A high expression of PDGFRb in the tumor stroma has been associated with unfavorable clinicopathological variables and shorter recurrence free and breast cancer specific survival, univariably, in a population-based cohort [11] although there are also studies which have failed to confirm the prognostic effect [12]. However, the application of a gene expression signature reflecting PDGFRb-activation stably indicated prognostic relevance of a high signature score for shorter recurrence free survival and/or breast cancer specific survival in four independent patient cohorts [13].

Stromal PDGFRb expression could also be treatment predictive. Higher expression of PDGFRb has been associated with a significantly decreased benefit from tamoxifen in ER-positive invasive breast cancer [14]. The mechanistic relationship has not yet been elucidated.

Thus, findings from these studies underline the importance of a study design that can discriminate prognostic and treatment related effects. Furthermore, these observations also point towards the complex interplay between PDGFRb as a potential marker of distinct stroma cell populations and at the same time as an active signaling receptor driving tumor progression.

Preclinical models of different solid tumor types have suggested potential mechanisms of how PDGF-activated stroma cells can modulate treatment effects and prognosis (reviewed in [9]). These mechanisms range from modulation of interstitial fluid pressure impairing drug uptake [15–17] to induction of a basal like tumor cell and promotion of dissemination through paracrine signaling [18–20]. With regards to RT, experimental models have provided evidence for general radioprotective effects of fibroblasts on cancer cells [21–23], but a role of PDGF signaling in these models has not yet been demonstrated.

As the current literature is conflicting regarding the function of stromal PDGFRb on prognosis as well as treatment response in invasive breast cancer, the purpose of the present study was to analyze the prognostic and predictive impact of stromal PDGFRb on ipsilateral breast tumor recurrence (IBTR), any recurrence and breast cancer specific death (BCSD) in a large and clinically well-annotated randomized RT trial of early stage breast cancer patients.

## Materials and methods

### Patient cohort

**Fig. 1:**
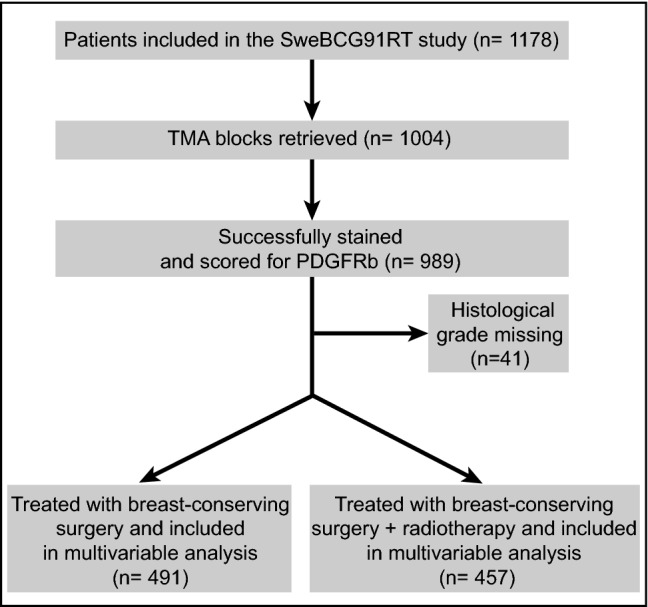
CONSORT flowchart. Patients from the Swedish Breast Cancer Group 91 Radiotherapy (SweBCG91RT) randomized radiotherapy trial included in the present biomarker study. *RT* radiotherapy, *TMA* tissue microarray, *PDGFRb* platelet derived growth factor receptor beta

The retrospective analysis included patients from the SweBCG91RT trial who have been described elsewhere [24, 25] (Table 1). In short, 1178 lymph node negative (N0) patients with stage I or IIA breast cancer were randomly assigned to BCS with or without whole breast RT between the years 1991 and 1997 and followed for a median time of 15.2 years (Fig. 1). Tumor blocks from the initial surgery were retrieved, and tumors were classified according to the St Gallen International Breast Cancer Conference Expert Panel 2013 using immunohistochemical panels.

**Table 1:** Distribution of clinicopathological variables in the SweBCG91RT cohort depending on PDGFRb score

Variables	PDGFRb-low	PDGFRb-medium	PDGFRb-high	*p* value*
Age (years)				
≤ 55	82 (26.9%)	117 (37.4%)	175 (47.2%)	
55	223 (73.1%)	196 (62.6%)	196 (52.8%)	< 0.001
Tumor size (mm)				
≤ 10	94 (35.3%)	63 (24.7%)	83 (25.8%)	
11–15	105 (39.5%)	105 (41.2%)	146 (45.3%)	
16–20	42 (15.8%)	62 (24.3%)	58 (18%)	
> 20	25 (9.4%)	25 (9.8%)	35 (10.9%)	0.037
Histological grade				
I	48 (16.5%)	42 (14%)	56 (15.7%)	
II	182 (62.5%)	189 (62.8%)	197 (55.3%)	
III	61 (21%)	70 (23.3%)	103 (28.9%)	0.13
Subtype				
Luminal A	192 (66.4%)	178 (58.6%)	180 (50.1%)	
Luminal B	67 (23.2%)	81 (26.6%)	110 (30.6%)	
HER2+	14 (4.8%)	20 (6.6%)	30 (8.4%)	
Triple negative	16 (5.5%)	25 (8.2%)	39 (10.9%)	0.004
ER status				
Negative	46 (15.1%)	54 (17.3%)	81 (21.8%)	
Positive	259 (84.9%)	259 (82.7%)	290 (78.2%)	0.066
Endocrine therapy				
No	279 (91.5%)	294 (93.9%)	345 (93%)	
Yes	26 (8.5%)	19 (6.1%)	26 (7%)	0.49
Chemotherapy				
No	304 (99.7%)	307 (98.1%)	360 (97%)	
Yes	1 (0.3%)	6 (1.9%)	11 (3%)	0.024
RT treatment				
No	159 (52.1%)	158 (50.5%)	194 (52.3%)	
Yes	146 (47.9%)	155 (49.5%)	177 (47.7%)	0.88

*Chi-square test or Fisher’s exact test

ER and HER2 evaluation has been described previously [25]. In brief, the cutoff used to consider a tumor ER positive was 1%, for PgR the cutoff was ≥ 20% to distinguish luminal A-like from luminal B-like tumors. Triple negative tumors were defined as negative for ER, PgR and HER2. HER2 was considered positive if 3+ on immunohistochemistry level or amplified on silver in situ hybridization [25]. Patients were well balanced regarding clinicopathological baseline characteristics across the treatment arms as shown previously [24].

### Immunohistochemistry (IHC)

The Ventana Benchmark Discovery autostainer system (NexES V10.6) was used for immunohistochemical staining of PDGFRb on 4 μm freshly cut sections from formalin-fixed paraffin embedded tissue microarray (TMA) blocks. The protocol included extended antigen retrieval with pH10 Tris buffer (Sigma-Aldrich and Merck Kgaa, Darmstadt, Germany) and incubation for 1 h at 37 °C with the primary antibody (rabbit monoclonal anti-PDGFRb antibody, clone 28E1, #3169 Cell Signaling, Danvers MA, US) diluted at 1:100 dilution in Discovery Antibody Diluent (Ventana, Tuscon, Arizona, US). Chromogenic detection was performed using the Discovery OmniMap anti-rabbit HRP (RUO) kit (Ventana) with secondary antibody incubation for 32 min at room temperature. Hematoxylin II was applied for 10 min and subsequent bluing for 6 min (Ventana) in order to obtain counterstaining. Antibody-based cross detection of the structurally related PDGFRa was excluded as described previously [26].

### Marker evaluation

**Fig. 2:**
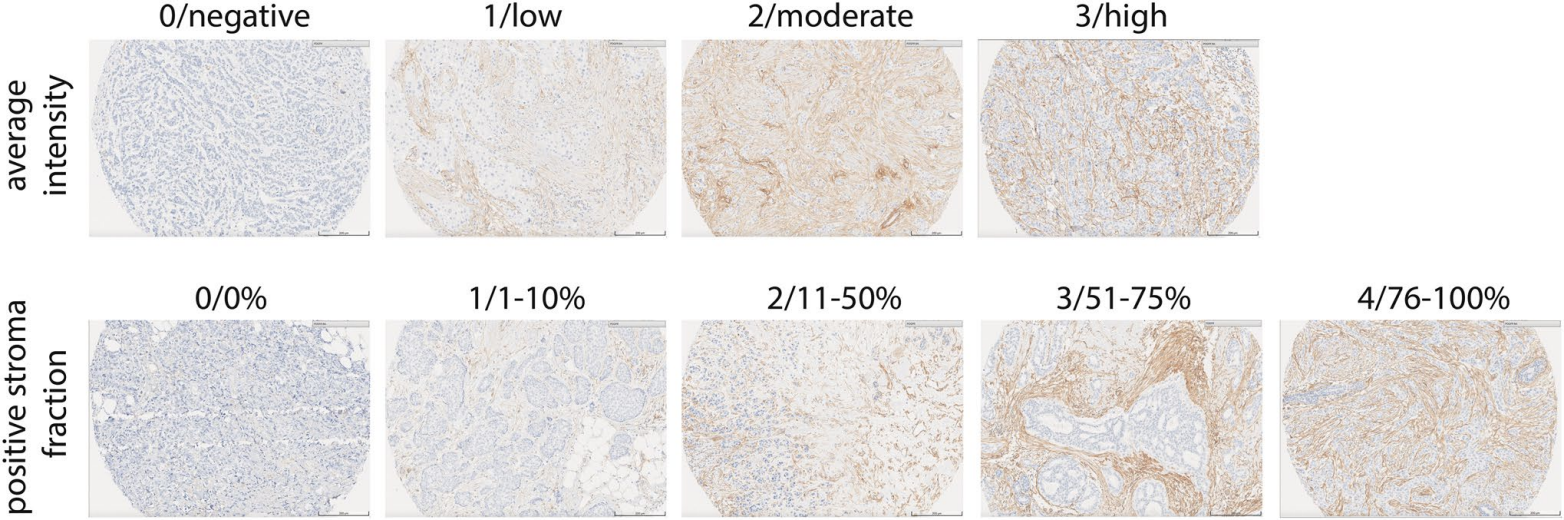
IHC staining and scoring of PDGFRb. Example pictures of PDGFRb expression detected by immunohistochemistry (IHC). Staining of PDGFRb was performed on tissue microarrays and evaluated by two independent raters for average intensity (0/negative; 1/low; 2/moderate; 3/high) and positive stroma fraction (0/0%; 1/1–10%; 2/11–50%; 3/51–75%; 4/76–100%). Scale bar represents 200 μm

The stained slides were scanned for evaluation (PathXL, Belfast, Northern Ireland). Scoring of stromal PDGFRb staining was performed blinded by two independent raters (CS and RJE) for average intensity following a four-grade scale (0/negative; 1/low; 2/moderate; 3/high) and positive stroma fraction as well as overall stroma abundance following a five-grade scale (0/0%; 1/1–10%; 2/11–50%; 3/51–75%; 4/76–100%) (Fig. 2). Furthermore, the overall stroma fraction was rated on a five-grade scale (0/0%; 1/1–10%; 2/11–50%; 3/51–75%; 4/76–100%). Tissue evaluation was guided by a breast pathologist (LAA). TMAs included two cores of 1.0 mm diameter per patient. The degree of scoring consistency between raters was evaluated using unweighted Cohen’s kappa (*κ*) correlation [27]. Rare cases for which the scores of the raters differed by more than two grades were reevaluated to exclude technical errors. The evaluations of both raters were averaged, and the product between PDGFRb staining intensity and positive stroma fraction was calculated. For the final analysis data was split in tertiles, as predefined, and referred to as PDGFRb low (*n* = 305), medium (*n* = 313) or high (*n* = 371) score group.

### Statistics

Time to IBTR as first event within 10 years was used as primary endpoint. Secondary endpoints were time to any breast cancer recurrence within 10 years (IBTR, regional recurrence or distant recurrence) and time to breast cancer specific death (BCSD) within 15 years. Regional recurrence, distant recurrence and death were considered competing risks for IBTR.

Known clinical variables were tested first in univariable and then, if significant, in multivariable analysis including age group, histological grade, subtype and RT treatment. Subtype was kept in multivariable analysis, despite not being significant in univariable analysis, because of the biologic relevance. Hazard ratios (HRs) were calculated with cause-specific Cox proportional hazards regression to reflect the biologic effect of RT in the presence of competing risks. Correlation analysis between clinicopathologic parameters and stromal PDGFRb status was tested using Spearman’s Rank test.

Figures of cumulative incidence were created according to the method by Fine and Gray [28]. *p* values for the hazard ratio between compared groups were denoted P_CIF_ in the plots. *p* values < 0.05 were considered significant. STATA 15.1 was used for analysis (StataCorp. 2017. Stata: Release 15. Statistical Software. College Station, TX: StataCorp LLC).

The proportional hazards assumption was checked graphically and tested with Schoenfeldt’s test. It was violated for RT, histological grade, subtype and RT: PDGFRb score and these values should thus be interpreted as the mean value over 10 years.

## Results

### Marker evaluation

Out of 1004 cases included in the TMA, 989 cases were successfully scored (Figs. 1, 2). Using Cohen’s kappa statistics, the inter-rater agreement was in the moderate range for scoring of the average staining intensity (*κ* = 0.59) and of the positive stroma fraction (*κ* = 0.45).

### Correlation with clinicopathological patient characteristics

**Fig. 3:**
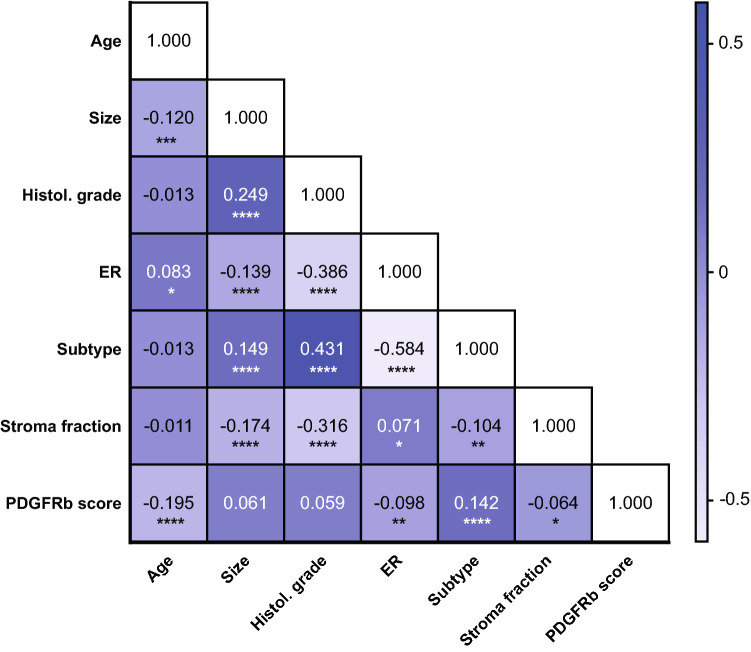
Correlation between PDGFRb and clinicopathologic parameters. Spearman’s Rank test-based correlation analysis between clinicopathologic parameters and stromal PDGFRb status in patients of the SweBCG91RT trial. PDGFRb score, age, tumor size and overall stroma fraction are included as continuous variables. Histological grade comprises grade I, II and III. Estrogen receptor (ER) status is classified as yes or no. Subtype refers to subtypes Luminal A-like, Luminal B-like, HER2 positive or triple negative. Numbers indicate Spearman’s *rho* (*ρ*); *p < 0.05; **p < 0.01; ***p < 0.001; ****p < 0.0001

The distribution of clinicopathological variables can be seen in Table 1. A high PDGFRb score was associated with ER negativity (Spearman’s *ρ* = 0.098, *p* = 0.003), young age (*ρ* = 0.195, *p* < 0.001), subtype (*ρ* = 0.142, *p* < 0.001) and a lower overall stroma fraction (*ρ* = 0.064, *p* = 0.043) in Spearman’s Rank tests (Fig. 3).

### Prognostic potential of stromal PDGFRb expression

**Fig. 4:**
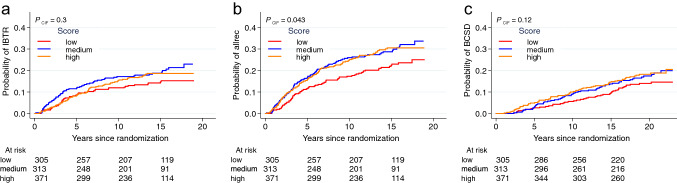
Prognostic impact of PDGFRb. Univariable analysis of cumulative incidence of ipsilateral breast tumor recurrence (IBTR, **a**), any recurrence (allrec, **b**) and breast cancer specific death (BCSD, **c**) in patients of different PDGFRb score groups. Red lines represent the PDGFRb low, blue the medium and orange the high score group. Tables indicate numbers of patients at risk. *p* values are based on the cumulative incidence function (CIF) numbers over ten years since breast conserving surgery

No prognostic impact was observed for any of the PDGFRb score groups with regards to IBTR at 10 years after BCS (Fig. 4a, Table 2). For any recurrence, a significantly increased risk was detected in univariable analysis for patients with a medium (HR 1.58, CI 95% 1.11–2.23, *p* = 0.011) or high PDGFRb score (HR 1.49, CI 95% 1.06–2.10, *p* = 0.021) as compared to the PDGFRb low score group (Fig. 4b, Table 2). In a multivariable analysis including histological grade, age, RT and subtype, the significance remained for the PDGFRb medium (HR 1.46, CI 95% 1.01–2.11, *p* = 0.042) but not the PDGFRb high score group (HR 1.32, CI 95% 0.93–1.88, *p* = 0.125) (Table 2). PDGFRb score was not significantly associated with risk of BCSD within 15 years from diagnosis (Fig. 4c, Table 2).

**Table 2:** Prognostic performance of PDGFRb score group in uni- and multivariable Cox regression analysis

Endpoint	PDGFRb score group	Univariable HR (95% CI); *p* value	Multivariable incl. RT, grade, age group, subtype HR (95% CI); *p* value
IBTR, 10 years	Low	1	1
	Medium	1.51 (0.99–2.30); 0.057	1.44 (0.92–2.62); 0.111
	High	1.33 (0.87–2.02); 0.187	1.20 (0.77–1.89); 0.423
Any recurrence, 10 years	Low	1	1
	Medium	1.58 (1.11–2.23); **0.011**	1.46 (1.01–2.11); **0.042**
	High	1.49 (1.06–2.10); **0.021**	1.32 (0.93–1.88); 0.125
BCSD, 15 years	Low	1	1
	Medium	1.37 (0.85–2.21); 0.191	1.24 (0.77–2.01); 0.381
	High	1.52 (0.96–2.38); 0.075	1.26 (0.79–2.01); 0.333

*p* values are based on Wald test; *p* values < 0.05 in bold text

*IBTR* ipsilateral breast tumor recurrence, *BCSD* breast cancer specific death, *RT* radiotherapy, *HR* hazard ratio, *CI* confidence interval

### RT-predictive potential of stromal PDGFRb expression

**Fig. 5:**
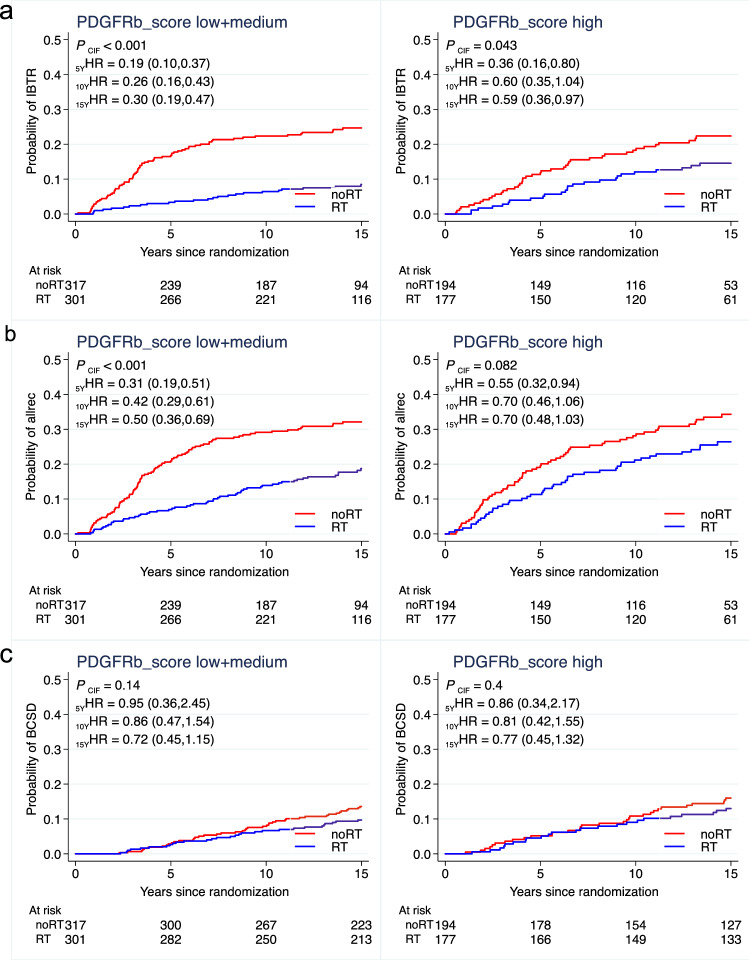
Radiotherapy response-predictive potential of stromal PDGFRb expression. Univariable analysis of cumulative incidence of ipsilateral breast tumor recurrence (IBTR, **a**), any recurrence (allrec, **b**) and breast cancer specific death (BCSD, **c**) with or without adjuvant radiotherapy (RT) in patients of different PDGFRb score groups. Red lines represent patients not receiving adjuvant RT treatment (no RT) and blue lines represent adjuvant RT treated patients. Tables indicate numbers of patients at risk. *p* values are based on the cumulative incidence function (CIF) numbers over ten years since breast conserving surgery. Hazard ratios (HR) are provided for 5, 10 and 15 year time points

The benefit of RT regarding the risk of IBTR was significant in univariable as well as multivariable analysis including histological grade, age and subtype for the PDGFRb low [univariable: HR 0.25, CI 95% 0.11–0.56, *p* < 0.001; multivariable: 0.29 (0.12–0.67), *p* = 0.004] and medium [univariable: HR 0.25, CI 95% 0.13–0.48, *p* < 0.001; multivariable: 0.31 (0.16–0.59), *p* < 0.001] score groups but not in the PDGFRb high [univariable: HR 0.61, CI 95% 0.35–1.05, *p* = 0.073; multivariable: 0.64 (0.36–1.11), *p* = 0.110] score group at 10 years after BCS (Fig. 5a, Table 3).

**Table 3:** Interaction between PDGFRb score and RT treatment in Cox regression analysis

Endpoint	PDGFRb score group	Univariable RT vs. non-RT HR (95% CI); *p* value	Multivariable RT vs. non-RT incl. grade, age group, subtype HR (95% CI); *p* value
IBTR, 10 years	Low	0.25 (0.11–0.56); *p* = **0.001**	0.29 (0.12–0.67); *p* = **0.004**
	Medium	0.25 (0.13–0.48); *p* < **0.001**	0.31 (0.16–0.59); *p* < **0.001**
	High	0.61 (0.35–1.05); *p* = 0.073	0.64 (0.36–1.11); *p* = 0.110
	Interaction PDGFRb score: RT		*p* = 0.153
Any recurrence, 10 years	Low	0.50 (0.28–0.89); *p* = **0.018**	0.57 (0.32–1.04); *p* = 0.067
	Medium	0.37 (0.23–0.60); *p* < **0.001**	0.46 (0.28–0.75); *p* = **0.002**
	High	0.70 (0.46–1.06); *p* = 0.089	0.75 (0.49–1.15); *p* = 0.192
	Interaction PDGFRb score: RT		*p* = 0.320
BCSD, 15 years	Low	1.05 (0.51–2.18); *p* = 0.888	1.12 (0.54–2.32); *p* = 0.768
	Medium	0.54 (0.29–1.00); *p* = 0.051	0.70 (0.35–1.26); *p* = 0.271
	High	0.77 (0.45–1.32); *p* = 0.338	0.88 (0.51–1.51); *p* = 0.633
	Interaction PDGFRb score: RT		*p* = 0.636

*p* values are based on Wald test; *p* values < 0.05 in bold text

*IBTR* ipsilateral breast tumor recurrence, *BCSD* breast cancer specific death, *RT* radiotherapy, *HR* hazard ratio, *CI* confidence interval

Likewise, the RT benefit regarding the risk for any recurrence was less pronounced in the PDGFRb high score group [univariable: HR 0.70, CI 95% 0.46–1.06, *p* = 0.089; multivariable: 0.75 (0.49–1.15), *p* = 0.192] as compared to the PDGFRb low [univariable: HR 0.50, CI 95% 0.28–0.89, *p* = 0.018; multivariable: 0.57 (0.32–1.04), *p* = 0.067] and medium [univariable: HR 0.37, CI 95% 0.23–0.60, *p* < 0.001; multivariable: 0.46 (0.28–0.75), *p* = 0.002] score groups.

No significant interaction between RT and PDGFRb score could however be detected for IBTR (*p* = 0.153) or any recurrence (*p* = 0.320) (Fig. 5b, Table 3). No benefit from RT regarding BCSD was observed for any of the PDGFRb score groups at 15 years after breast conserving surgery and no significant interaction between PDGFRb score and RT was noted for BCSD (*p* = 0.636) (Fig. 5c, Table 3).

## Discussion

Our study suggests that patients with higher expression of PDGFRb might have an increased risk of any breast cancer recurrence, but due to correlation with younger age and ER negativity, a function of PDGFRb as independent prognostic marker could not be demonstrated. Furthermore, our analyses demonstrated, both univariably as well as multivariably, that patients of the high PDGFRb score group derive less benefit from adjuvant RT in terms of IBTR as compared to the low and medium score groups. However, since the interaction test between PDGFRb and RT was not significant, our data does not confirm stromal PDGFRb expression as a predictive biomarker for RT benefit in early stage invasive breast cancer.

PDGFRb is a key regulator of fibroblasts and mural cells and has been previously suggested, both by functional and correlative studies, to play a role in the progression and treatment response of invasive breast cancer [8, 9, 11, 14, 29]. However, published findings are partly conflicting most likely due to study designs not allowing a clear discrimination of prognostic and treatment related effects. In this study we analyzed the prognostic and predictive impact of stromal PDGFRb in the randomized SweBCG91RT trial.

PDGFRb has previously been shown to correlate with unfavorable clinicopathological variables such as ER negativity, younger age and higher histological grade [11, 14]. These associations were confirmed in our study and could explain part of the prognostic effect of PDGFRb expression. However, the prognostic influence remained significant in multivariable analysis regarding any recurrence for the PDGFRb medium score group patients, which indicates that PDGFRb can provide independent prognostic information. In the present study, a tendency towards higher IBTR risk among patients with higher PDGFRb expression was also noted, although these results were not significant. These results are in line with previous reports describing a similar association between high stromal PDGFRb expression and shorter time to recurrence in a population-based cohort including both patients with negative and positive nodal status as well as patients with and without adjuvant endocrine treatment, chemotherapy or the combination [11].

PDGFRb is mainly expressed on fibroblasts and vascular mural cells, and both stromal cell types are a key source of growth factors and cytokines [9, 10]. The secretome of cancer associated fibroblasts (CAFs) has been connected to therapy resistance in breast cancer [19, 30–33]. In addition, CAFs can affect other cells of the tumor microenvironment such as immune cells and vascular cells and thereby indirectly influence tumor progression and therapy efficacy (reviewed in [34–36]). CAFs have been linked to immunosuppression, mainly by inhibiting T cell infiltration and activation [37–41], and tumor-infiltrating lymphocytes (TILs) were demonstrated to provide prognostic and treatment predictive information in breast cancer [3, 42, 43]. Potential prognostic and predictive effects of stromal PDGFRb expression in the primary tumor could be mediated by paracrine acting factors released by the microenvironment which act directly or indirectly on the tumor to promote progression and render tumor cells insensitive to RT.

Activation of PDGFRb, in particular on fibroblasts, has also been demonstrated to induce an upregulation of hepatocyte growth factor (HGF) and stanniocalcin-1 (STC1), with the latter having furthermore been linked to increased distant metastasis in several murine cancer models [18, 44–46]. In our study, the medium and high PDGFRb groups showed an increased propensity for any recurrence in univariable analysis, while no significant differences in rate of IBTR only were observed between the groups.

In invasive breast cancer, a comprehensive IHC analysis approach recently identified four functional different fibroblast subsets [38], of which one subset was high in fibroblast activation protein (FAP) and PDGFRb expression and functionally linked to immunosuppression and pro-invasive effects [37, 38]. Another subset, defined by CD29, alpha smooth muscle actin (ASMA) and also PDGFRb expression was assigned to pro-metastatic effects mostly through matrix remodeling [37]. A specific functional role of PDGFRb expression was however not identified within these studies and it is unclear if the prognostic or potentially predictive effects are mediated directly by PDGFRb expressing cells through downstream-signaling or indirectly through an effect on other cells of the tumor microenvironment, such as TILs. In addition, PDGFRb could simply be a marker for a functional fibroblast or vascular mural cell subset with distinct effects on tumor progression. However, PDGFRb did not correlate positively with the overall stroma fraction. In our cohort, the overall stroma fraction was highest among Luminal A tumors and lowest among triple negative tumors. PDGFRb score showed the opposite distribution among subtypes and was instead correlated with unfavorable clinicopathological variables. We believe this can explain why PDGFRb and overall stroma fraction did not correlate. Previous studies have shown that a higher stroma fraction is associated with an unfavorable prognosis, particularly in triple negative tumors [47]. However, among ER positive tumors a higher stroma content has also been associated with favorable clinicopathological variables and with a better prognosis which conforms with our findings [48, 49]. PDGFRb could simply be a marker for a functional fibroblast or vascular mural cell subset with distinct effects on tumor progression, and our study suggests that it may not correlate strongly with overall stroma. Recent single-cell sequencing data of stroma cells from a murine breast cancer model suggested the existence of specific vascular and matrix-remodeling CAF subsets [50]. It would therefore be of interest to further relate stromal PDGFRb-positivity to vessel density as well as abundance and composition of extracellular matrix. Additionally, studies applying multiplexed panels of markers for fibroblasts and pericytes as well as their activation status could provide a more specific definition of mesenchymal cell subsets as well as cell type stratification and thereby refine the findings of the presented study.

A strength of the presented study is the large patient number and the randomized design of the cohort allowing investigation of prognostic and predictive effects differentially. However, the study is to a certain extent limited through the utilization of a TMA format, which, although two cores per patient are included, may not sufficiently reflect heterogenous PDGFRb expression throughout the tumor tissue. Furthermore, PDGFRb scoring was performed manually by two independent raters and despite a moderate interrater agreement, unbiased digital approaches may be more sensitive. Initial digital approaches have been described [26, 51, 52] but are still under refinement given the fact that especially in early stage breast cancers, normal tissue regions very often are present and need to be excluded. Of note, in this retrospective study, the patients were categorized into PDGFRb score groups based on tertiles as a predefined cut-off. However, future studies using optimized cut-off strategies and independent validation cohorts would be highly warranted to further study the potential RT-predictive nature of PDGFRb expression.

In summary, our study suggests that higher stromal PDGFRb expression is associated with an increased risk of any recurrence, which however can partly be explained by its association with estrogen receptor negativity and young age. Although a reduced RT benefit among patients with high PDGFRb was observed both in uni- as well as multivariable analysis, the interaction between PDGFRb and RT was not significant. Overall, the presented data motivates the experimental investigation of paracrine signaling initiated through stromal PDGFRb expression on tumor progression and resistance to RT.

## Data Availability

The datasets used and/or analyzed during the current study are available from the corresponding author on reasonable request.
